# Transcriptome sequencing of garlic reveals key genes related to the heat stress response

**DOI:** 10.1038/s41598-024-66786-4

**Published:** 2024-07-10

**Authors:** Qing-Qing Yang, Feng Yang, Can-Yu Liu, Yong-Qiang Zhao, Meng-Yao Li, Xin-Juan Lu, Jie Ge, Bi-Wei Zhang, Meng-Qian Li, Yan Yang, Ji-De Fan

**Affiliations:** 1 Xuzhou Institute of Agricultural Sciences in Jiangsu Xuhuai Area, Key Laboratory of Biology and Genetic Breeding of Sweetpotato, Ministry of Agriculture and Rural Affairs, Xuzhou, 221131 China; 2 College of Horticulture, Sichuan Agricultural University, Chengdu, 611130 China

**Keywords:** *Allium sativum* L., RNA-seq, Gene expression, Heat stress, Transcript profiles, Plant breeding, Plant molecular biology, Plant stress responses

## Abstract

With global warming, heat stress has become an important factor that seriously affects crop yield and quality. Therefore, understanding plant responses to heat stress is important for agricultural practice, but the molecular mechanism of high-temperature tolerance in garlic remains unclear. In this study, ‘Xusuan No. 6’ was used as the experimental material. After heat stress for 0 (CK), 2 and 24 h, transcriptome sequencing was used to screen metabolic pathways and differentially expressed genes (DEGs) closely related to heat stress and was further verified by quantitative real-time polymerase chain reaction (qRT-PCR). A total of 86,110 unigenes obtained from the raw transcriptome sequencing data were spliced. After 2 h of heat treatment, the expression levels of 8898 genes increased, and 3829 genes were decreased in leaves. After 24 h, the expression levels of 7167 genes were upregulated, and 3176 genes were downregulated. Gene Ontology enrichment analysis showed that DEGs were mainly enriched in seven categories: cellular processes, metabolic processes, binging, catalytic activity, cellular anatomical entity and protein-containing complex response to stimulus. Kyoto Encyclopedia of Genes and Genomes pathway enrichment showed that DEGs are involved in protein processing in the endoplasmic reticulum, plant hormone signal transduction, phenylpropanoid biosynthesis, and photosynthetic antenna proteins. Six genes were selected and further verified by qRT-PCR. In this study, the full-length transcriptome of garlic was constructed, and the regulatory genes related to the heat resistance of garlic were studied. Taken together, these findings can provide a theoretical basis for the cloning of heat resistance genes in garlic and for the analysis of heat resistance mechanisms.

## Introduction

Garlic (*Allium sativum* L.) is an important cash crop belonging to the Allium family. Moreover, garlic is a cold-loving crop with poor adaptability to high temperatures, and this seriously affects the growth and development of garlic, leading to a decline in its yield and quality^1,2^.

With changes in the global climate, heat stress has become one of the major environmental stressors limiting plant metabolism and crop productivity^3,4^. Studies have shown that heat stress can negatively affect plant growth, physiological processes, and metabolism^5^. Therefore, to cope with the damage caused by high temperatures, plants have formed complex physiological and biochemical adaptation mechanisms in the long-term evolution process to clear or repair denatured proteins and other biological macro-molecules and maintain cell homeostasis^6,7^. High temperatures usually damage proteins in the endoplasmic reticulum (ER), chloroplasts and cytoplasm^8^. Endoplasmic reticulum (ER) stress is a stress response caused by the accumulation of unfolded proteins in the ER lumen at high temperatures^9^. Some important regulatory factors have been found to help translate and fold proteins in the ER and cytoplasm, thereby regulating protein homeostasis and alleviating heat damage^10^. Liu et al. identified two types of unfolded protein response (UPR) signaling pathways in plants: one uses two transmembrane alkaline bright basic-leucine zipper transcription factors (bZIP17 and bZIP28), and the other involves a double protein kinase (RNA splicing factor IRE1) and its target RNA (bZIP60)^11^_._ In addition, plant hormones, as signaling molecules, are widely involved in plant responses to abiotic stress^12,13^. Abscisic acid (ABA), salicylic acid (SA), auxin/indoleacetic acid (IAA), and other plant hormones are also involved in the response of plants to high-temperature stress^14^. Salicylic acid can remove hydrogen peroxide produced by cucumber under high-temperature stress, thereby improving the heat resistance of cucumber^15^. The jasmonate signaling pathway is involved in the molecular regulation mechanism of plant response to high-temperature stress and provides an important gene resource for wheat heat tolerance molecular breeding^16^.

RNA-sequencing (RNA-seq) technology has been widely used in the study of the plant response to abiotic stress, including heat stress in maize (*Zea mays* L.) seedlings^17^, heat stress in eggplant leaves^18^, and drought stress in peanut seedlings^19^_._ Wang et al. performed transcriptome sequencing in garlic variety ‘Cangshan Siliuban’ and found that genes involved in hormone signaling and cell wall remodeling play an important role in garlic response to high salt stress^20^_._ These RNA-seq technologies have been widely used in the study of abiotic plant reactions, and many stress-response genes related to different metabolic processes have been identified, enriching information on the plant stress regulation network. However, there are few reports on the response genes and heat resistance mechanism of garlic to high-temperature stress. In this study, to analyze the molecular mechanism of response and adaptation of garlic seedlings to high-temperature stress, we exposed garlic seedlings to a high-temperature environment and conducted transcriptome sequencing to study the response genes at different time points of garlic high-temperature stress, providing a theoretical basis for the analysis of the high-temperature tolerance mechanism in garlic.

## Results

### Physiological response of garlic under high temperatures

**Figure 1:**
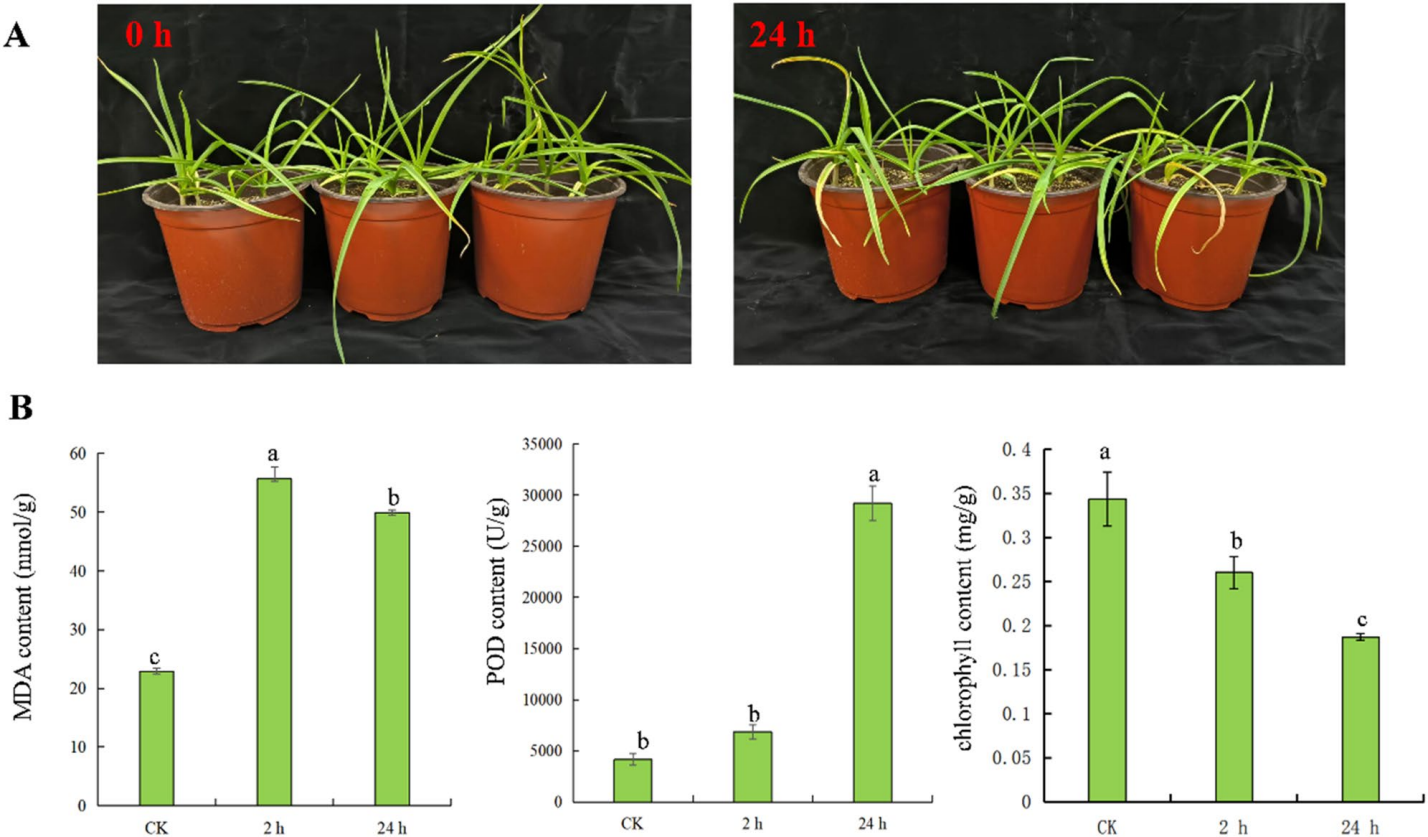
Phenotypic and physiological analysis of changes in garlic under heat stress. (**A**) Plant phenotype of garlic under heat temperature for 0 (CK) and 24 h; (**B**) Measurement of physiological indicators; different letters indicate significant difference between treatments at the 5% probability level, the same as below.

Under high-temperature stress, the leaves of garlic wilted after 24 h of heat stress (Fig. 1A). As shown in Fig. 1B, membrane peroxidation produced malondialdehyde (MDA) in large quantities, and the activity of MDA decreased gradually after 24 h but was maintained at a high level. The activity of peroxide (POD) increased rapidly after 2 h of high-temperature stress, indicating that the reactive oxygen scavenging system responded rapidly. With continuous exposure to a high temperature, the POD reached its peak at 24 h. High-temperature treatment significantly affects the physiological process of photosynthesis and ultimately leads to an increase or decrease in chlorophyll content. After high-temperature treatment, the chlorophyll content of garlic leaves gradually decreased.

### Assembly and functional annotation

**Figure 2:**
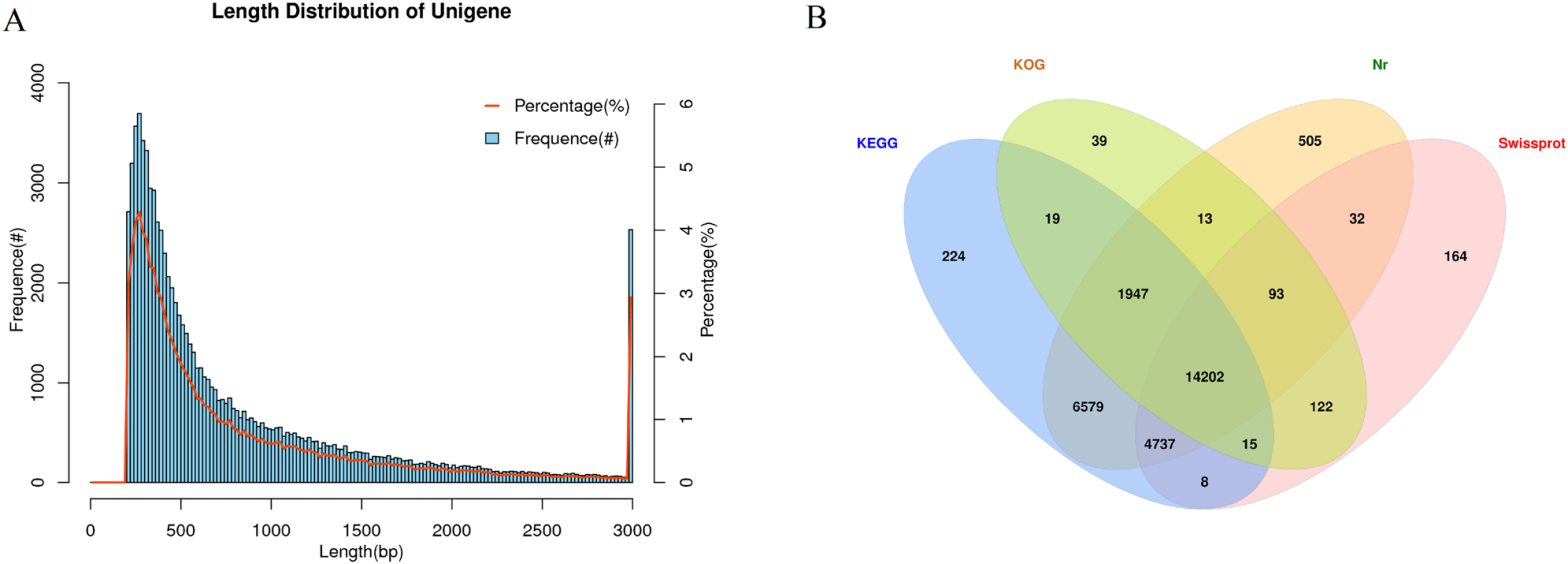
(**A**) Length distribution of unigene in the garlic transcriptome. (**B**) Venn diagram of the number of unigenes annotated in NR, KEGG, KOG, and Swiss-Prot database.

As shown in Fig. 2A, 86,110 unigenes were obtained, with an average length of 849 bp and 38.58% GC content. All Unigenes were annotated in the Non-Redundant Protein Database (Nr), KOG, Kyoto Encyclopedia of Genes and Genomes (KEGG), and Swiss-Prot databases, and the annotation rates were 32.6%, 19.10%, 32.20%, and 22.49%, respectively (Fig. 2B). Transcriptome sequencing quality analysis showed that multiple high-quality base sequences were obtained in the control group (CK) and after high-temperature treatment for 2 and 24 h, with Q20 and Q30 reaching more than 97% and 93%, respectively, and GC% reaching more than 40%, indicating that the transcriptome sequencing data in this study were of high quality and could be used for further research (Table 1).

**Table 1:** Base information statistics.

Samples	Raw data (bp)	Clean data (bp)	Q20%	Q30%	GC%
CK-1	9,374,447,100	9,293,798,421	97.82	93.54	45.28
CK-2	9,515,357,100	9,448,595,462	97.92	93.76	45.40
CK-3	12,518,945,700	12,425,907,821	98.00	93.94	45.40
T2-1	8,125,378,500	8,061,664,045	97.53	92.90	45.38
T2-2	10,061,572,500	9,985,417,113	97.87	93.70	45.28
T2-3	10,150,270,800	10,059,856,996	97.41	92.63	45.32
T24-1	9,763,164,000	9,681,843,974	97.89	93.69	45.23
T24-2	12,527,420,400	12,422,850,017	97.93	93.83	45.28
T24-3	11,195,277,900	11,086,657,071	97.39	92.58	45.24

### Functional classification

**Figure 3:**
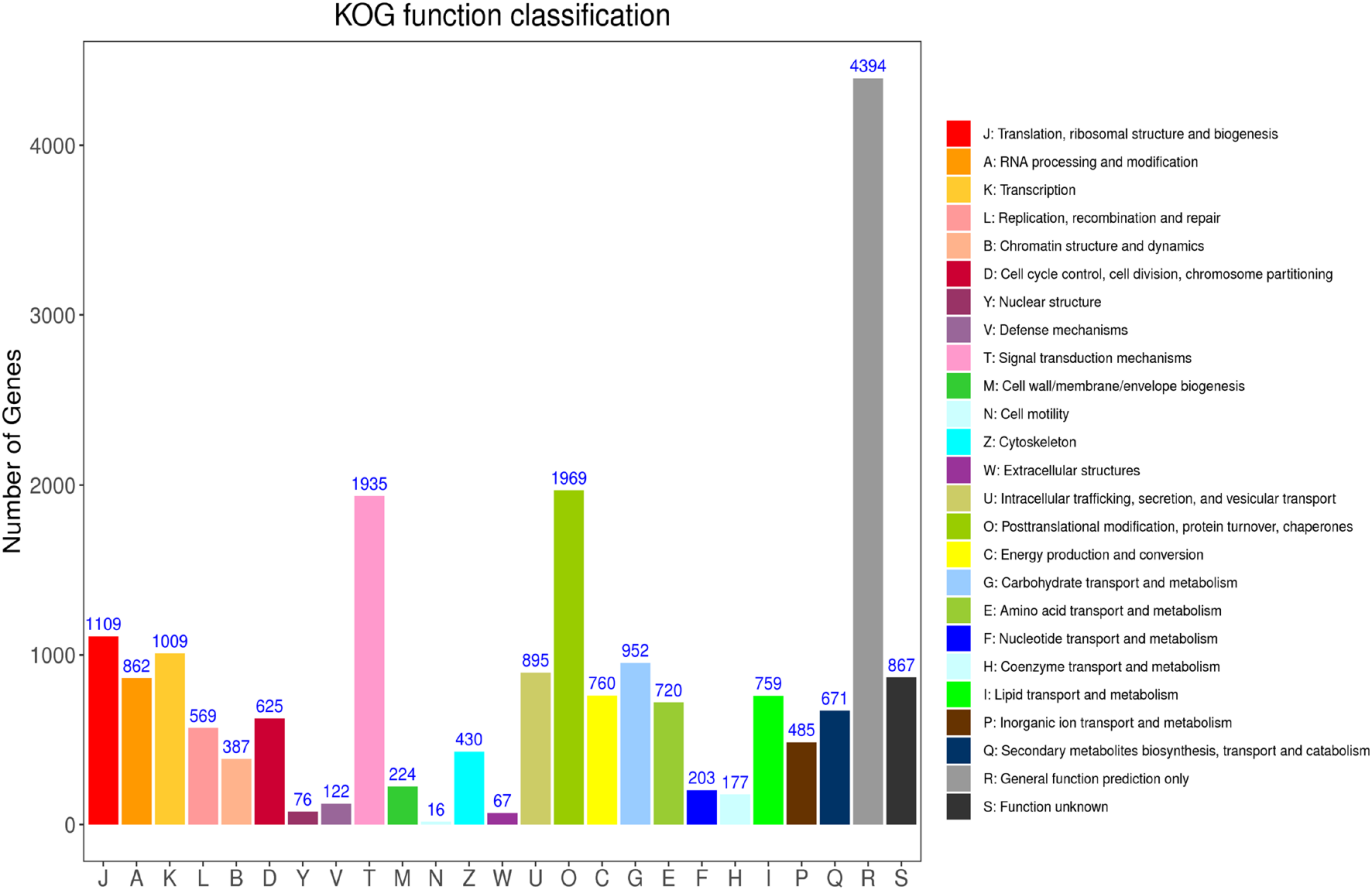
KOG function classification of unigenes in the garlic transcriptome.

A total of 42,483 unigenes were grouped into 26 functional categories (Fig. 3). Among them, cluster “general function prediction only” was the largest group, followed by “posttranslational modification, protein turnover, chaperones,” “signal transduction mechanisms,” and “translation ribosomal structure and biogenesis.”

**Figure 4:**
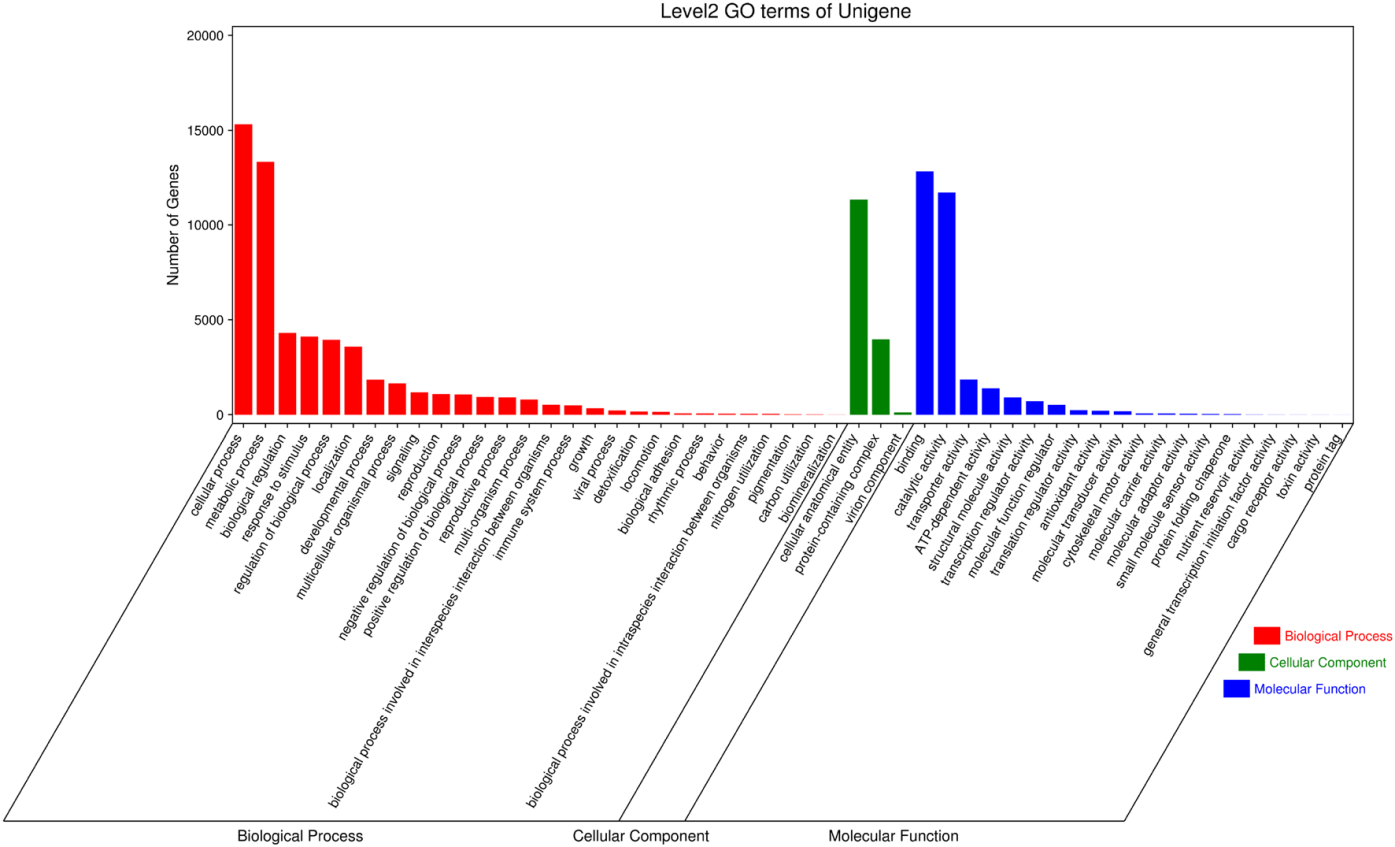
GO analysis of unigenes identified in the garlic transcriptome.

Through the analysis of DEGs in garlic exposed to high-temperature treatment, it was found that they were primarily concentrated in stress-related Gene Ontology (GO) annotations and KEGG pathways (Fig. 4). GO analysis of DEGs showed that the differences were mainly concentrated in biological processes, cell components and molecular functions. In the molecular function category, “binding” and “catalytic activity” were the most dominant classes, followed by “transporter activity” and “structural molecule activity. In the cellular process category, “cellular anatomical entity” and “protein-containing complex” were the most highly represented groups. After high-temperature stress treatment, metabolic processes and cellular processes play an important role in biological processes.

**Figure 5:**
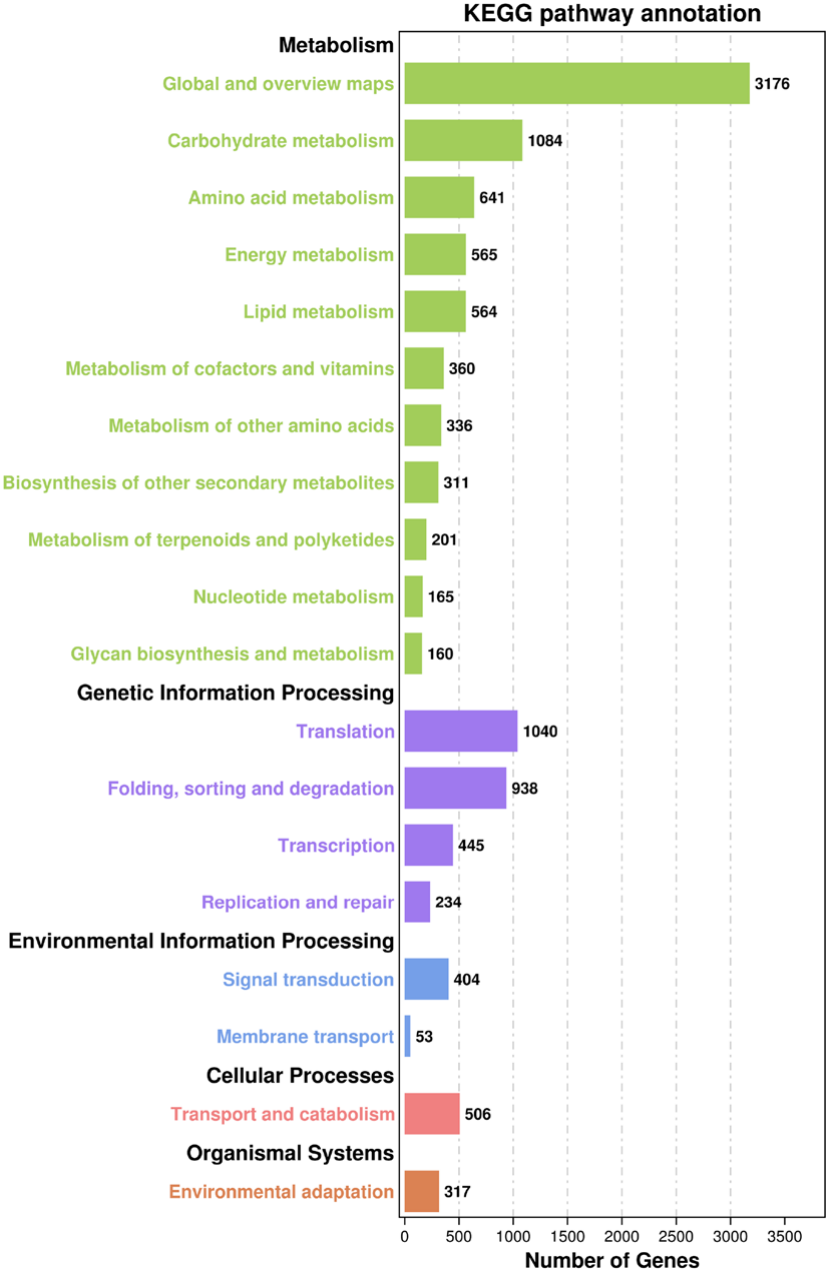
KEGG classification of unigenes in the garlic transcriptome.

KEGG enrichment demonstrated the metabolic pathways and bioinformatics functions of DEGs, as shown in Fig. 5. In this study, 2777 unigenes were distributed to five groups. The largest subgroup was “Global and overview maps,” followed by “carbohydrate metabolism,” which both belonged to the metabolism category.

### Transcriptional response of garlic seedlings to heat stress

**Figure 6:**
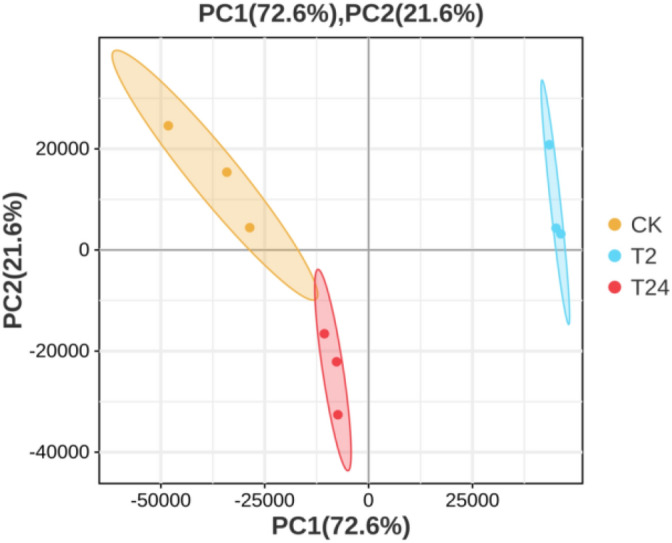
Principle component analysis plot of samples of garlic seedlings grown under normal conditions or exposed to heat stress. CK represents the control seedlings, while T2 and T24 represent heat-stressed seedlings.

Principal component analysis (PCA) was used to determine the similarity of gene expression in CK and garlic leaf samples after 2 and 24 h under high-temperature treatment (Fig. 6). Gene expression was significantly divided into three principal components in garlic seedlings under control and heat stress conditions. The first principal component accounted for 72.6% and the second for 21.6%.

**Figure 7:**
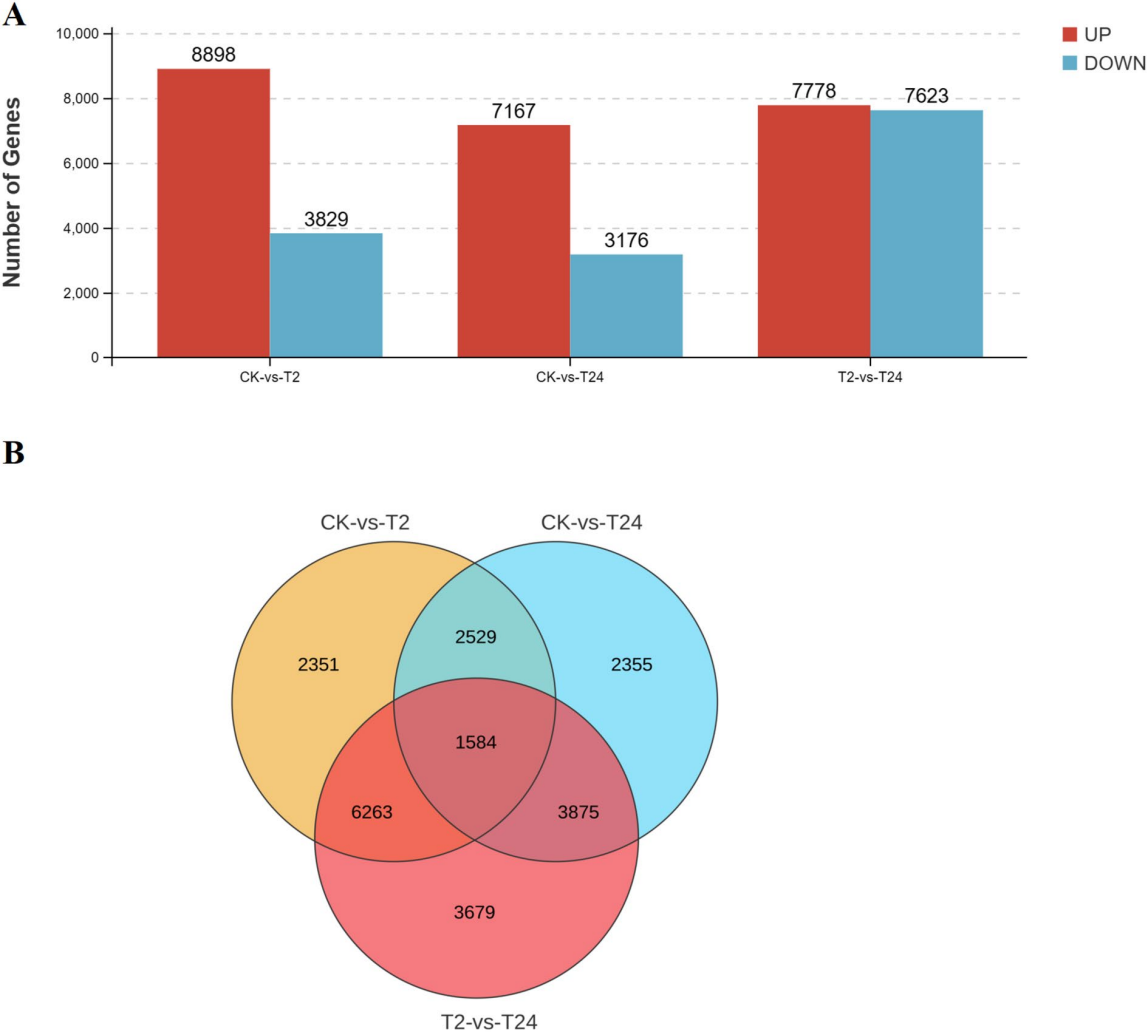
Analysis of differential gene expression analysis.

The transcription data of garlic treated with high-temperature stress for 2 and 24 h were compared with those of the control, and the expression levels of each gene in the four samples were compared and filtered with |log_2_ FC|≥ 1 and FDR < 0.05. There were 12,727, 10,343 and 15,401 up- or downregulated unigenes detected in the “CK- versus -T2,” “CK- versus -T24,” and “T2 versus T24” comparisons, respectively (Fig. 7A). The comparisons revealed that the three groups shared 1584 different genes (Fig. 7B).

### GO analysis of DEGs

**Figure 8:**
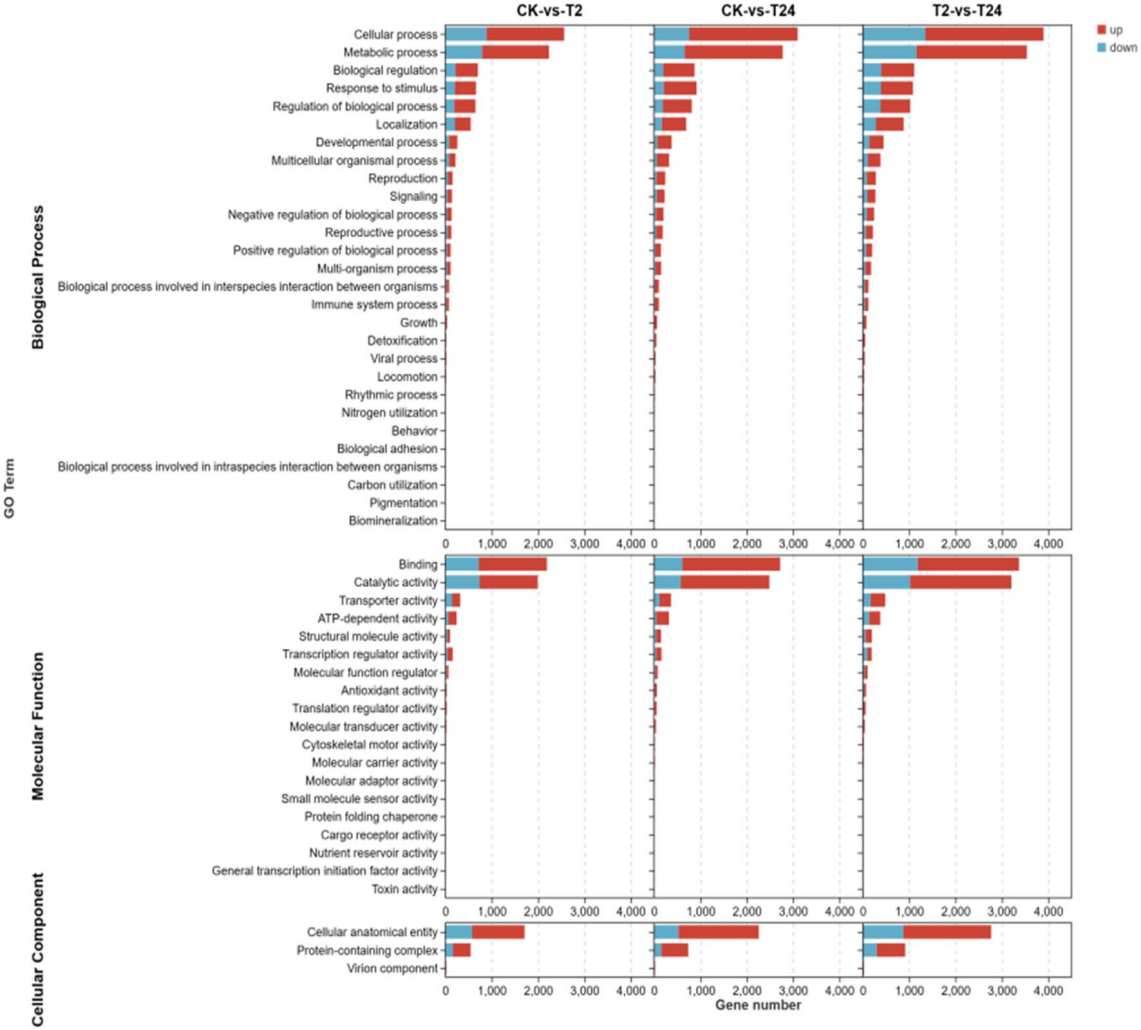
Histogram of GO classification of annotated genes.

Based on GO annotation analysis, DEGs were divided into three categories—biological process, cell component, and molecular function—with 50 different classification groups (Fig. 8). As shown in Table 2, regarding biological processes, DEGs were mainly concentrated in cellular processes, metabolic processes, biological regulation, and responses to stimuli. With respect to molecular functions, DEGs were mainly concentrated in binging, catalytic activity, and transporter activity. As cell components, DEGs were mainly concentrated in cellular anatomical entities and protein-containing complexes in response to stimuli. In addition, with the increase in high-temperature stress, the number of upregulated DEGS increased significantly, and the overall number of DEGS also increased. Therefore, we hypothesize that garlic may respond to high-temperature stress by affecting the expression levels of relevant DEGs at the transcriptional level.

**Table 2:** GO enrichment analysis of the DEGs.

	GO ID	GO Term	CK vs T2		CK vs T24	
			Up	Down	Up	Down
Biological processes	GO:0009987	Cellular process	1679	884	2342	755
	GO:0008152	Metabolic process	1440	796	2117	658
	GO:0065007	Biological regulation	484	218	676	197
	GO:0050896	Response to stimulus	457	203	711	206
	GO:0050789	Regulation of biological process	458	192	631	182
	GO:0051179	Localization	342	204	528	164
	GO:0032502	Developmental process	182	76	311	68
	GO:0032501	Multicellular organismal process	141	77	263	61
	GO:0000003	Reproduction	102	53	192	49
Molecular functions	GO:0005488	Binging	1473	118	2109	611
	GO:0003824	Catalytic activity	1255	738	1913	575
	GO:0005215	Transporter activity	176	143	250	114
	GO:0140657	ATP-dependent activity	179	63	273	47
	GO:0005198	Structural molecule activity	63	37	109	41
	GO:0140110	Transcription regulator activity	120	39	120	39
Cell components	GO:0110165	Cellular anatomical entity	1131	578	1732	528
	GO:0032991	Protein-containing complex	384	161	581	155

### KEGG analysis of DEGs

**Figure 9:**
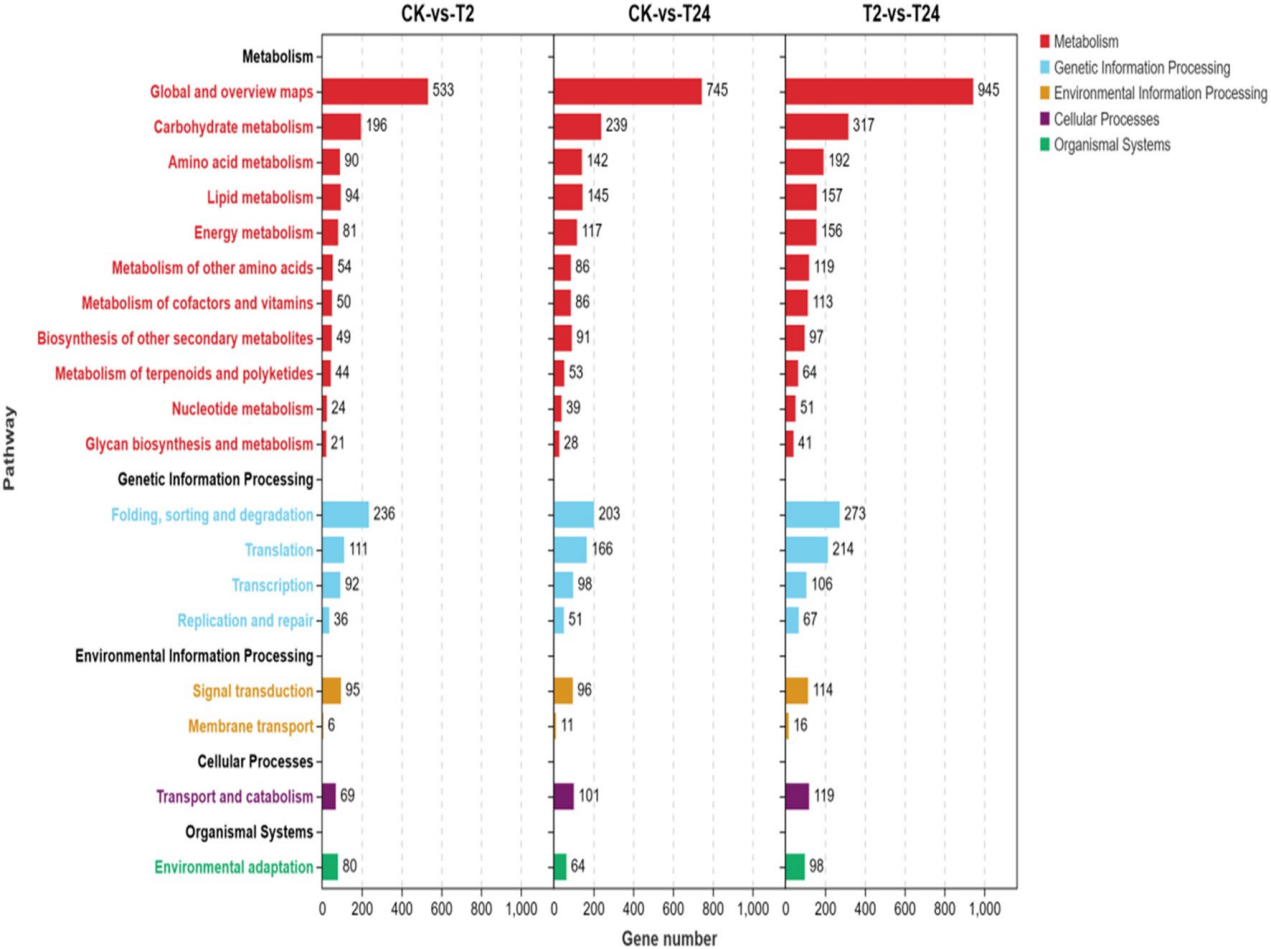
KEGG function analysis of differentially expressed genes.

KEGG enrichment is used to demonstrate the metabolic pathways and bioinformatics functions of differential genes. KEGG enrichment of DEGs in this study was divided into 5 KEGG pathway branches according to their metabolic and signaling pathways, including metabolism, genetic information processing, environmental information processing, cellular process and organismal systems (Fig. 9). In the “CK -versus -T2” comparison, 1961 DEGs were enriched into 130 pathways, among which 1236, 475, 101, 69, and 80 DEGs were involved in metabolism, genetic information processing, environmental information processing, cell process and organic system, respectively. In the “CK- versus -T24” comparison, 2561 DEGs were enriched into 130 pathways, among which 1771, 518, 107, 101, and 64 DEGs were involved in metabolism, genetic information processing, environmental information processing, cell process and organic system pathway branches, respectively. In the “T2- versus -T24” comparison, 3259 DEGs were enriched into 130 pathways, among which 2252, 660, 130, 119, and 98 DEGs were involved in metabolism, genetic information processing, environmental information processing, cell process and organic system pathway branches, respectively. In the three comparison weights, the DEGs involved in metabolic pathways were the most involved, and the DEGs involved in cellular process pathway branches were the least involved. In addition, with the extension of high-temperature stress exposure, DEGs in the organic system of the “CK- versus -T24” group decreased, and DEGs in the other four KEGG pathway branches gradually increased, especially in metabolic pathways.

### KEGG pathway analysis of DEGs

Among the top 10 KEGG pathways in the CK versus T2 comparison group, protein processing in the ER (ko04141) was annotated to 155 DEGs, accounting for 13.56% of the total. This was followed by plant–pathogen interaction (ko04626) and plant hormone signal transduction (ko04075), which were annotated in 70 (6.12%) and 63 (5.51%) DEGs, respectively (Table S1). In the comparison group of CK- versus -T24, protein processing in the ER (ko04141) was annotated in 125 DEGs in the first 20 pathways, accounting for 8.7% of the total, followed by phenylpropanoid biosynthesis (ko00940) and photosynthesis–antenna proteins (Ko00196), which were annotated in 38 (2.65%) and 10 (0.70%) DEGs, respectively (Table S2). In the T-2 versusT24 comparison group, protein processing in the ER (ko04141), which was annotated in 164 DEGs in the first 20 pathways, accounted for 9.1% of the total, followed by metabolic pathways (ko01100) and glutathione metabolism (ko00480), which were annotated in 912 (50.58%) and 50 (2.77%) DEGs, respectively (Table S3).

**Figure 10:**
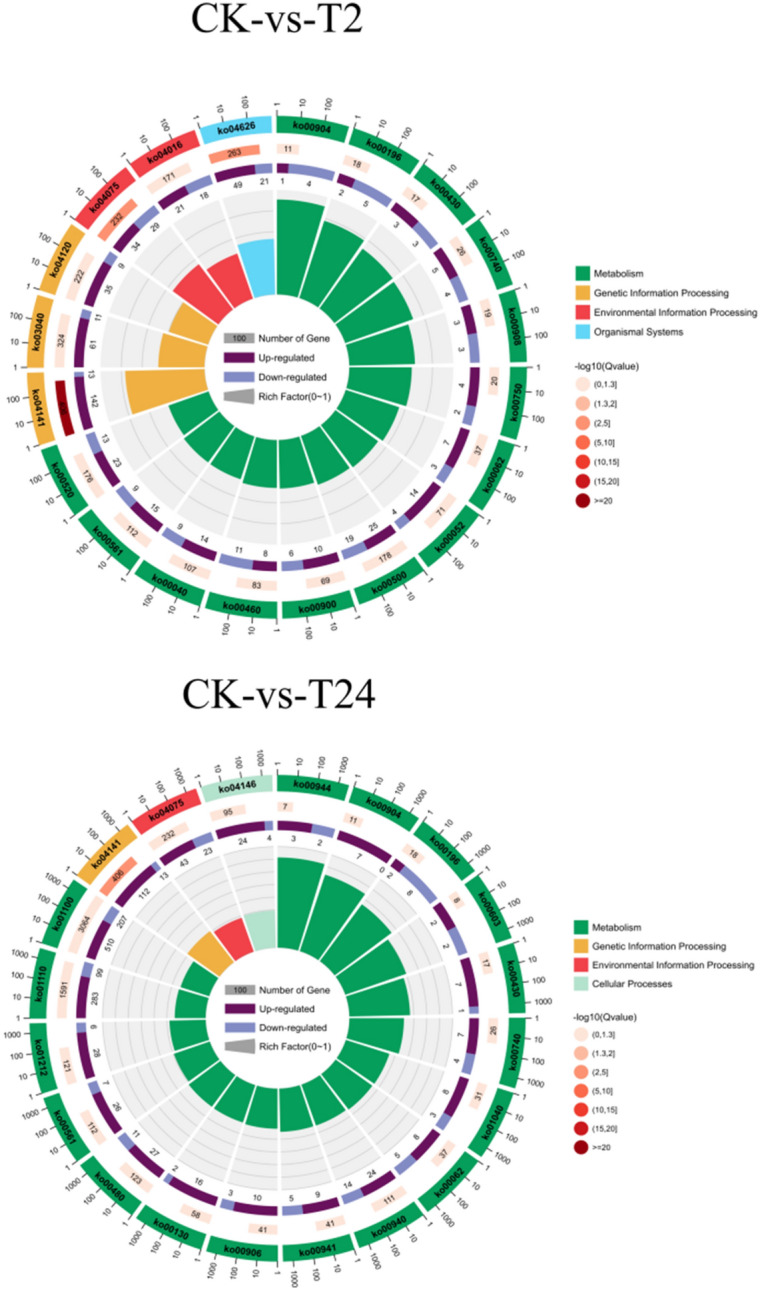
Top 20 KEGG pathways in garlic leaves under heat stress. The first and outer circles show the top 20 KEGG pathways that were enriched, while the scale outside the circle indicates the number of genes. Different colors represent different ontologies. Next, the KEGG pathway number in the background gene, along with the Q value, can be seen in the middle circles. Dark colors indicate genes that are upregulated, and light colors indicate genes that are downregulated. Below is a display of the specific value. The inner circle: rich factor).

In the CK versus T2 and CK versus T24 comparison groups, eight pathways were found to be the same, including protein processing in the ER (ko04141), plant hormone signal transduction (ko04075), photosynthesis-antenna proteins (ko00196), riboflavin metabolism (ko00740), diterpenoid biosynthesis (ko00904), taurine and hypotaurine metabolism (ko00430), fatty acid metabolism (ko00062) and glycerolipid metabolism (ko00561) (Fig. 10). Among these, the protein processing in the ER (ko04141) and plant hormone signal transduction (ko04075) pathways were significantly enriched by DEGs. KEGG pathway enrichment analysis showed that protein processing in the ER was the most significantly enriched pathway, suggesting that it plays a central role in garlic under high-temperature stress.

### Protein processing in ER-related genes under heat stress: expression change

**Figure 11:**
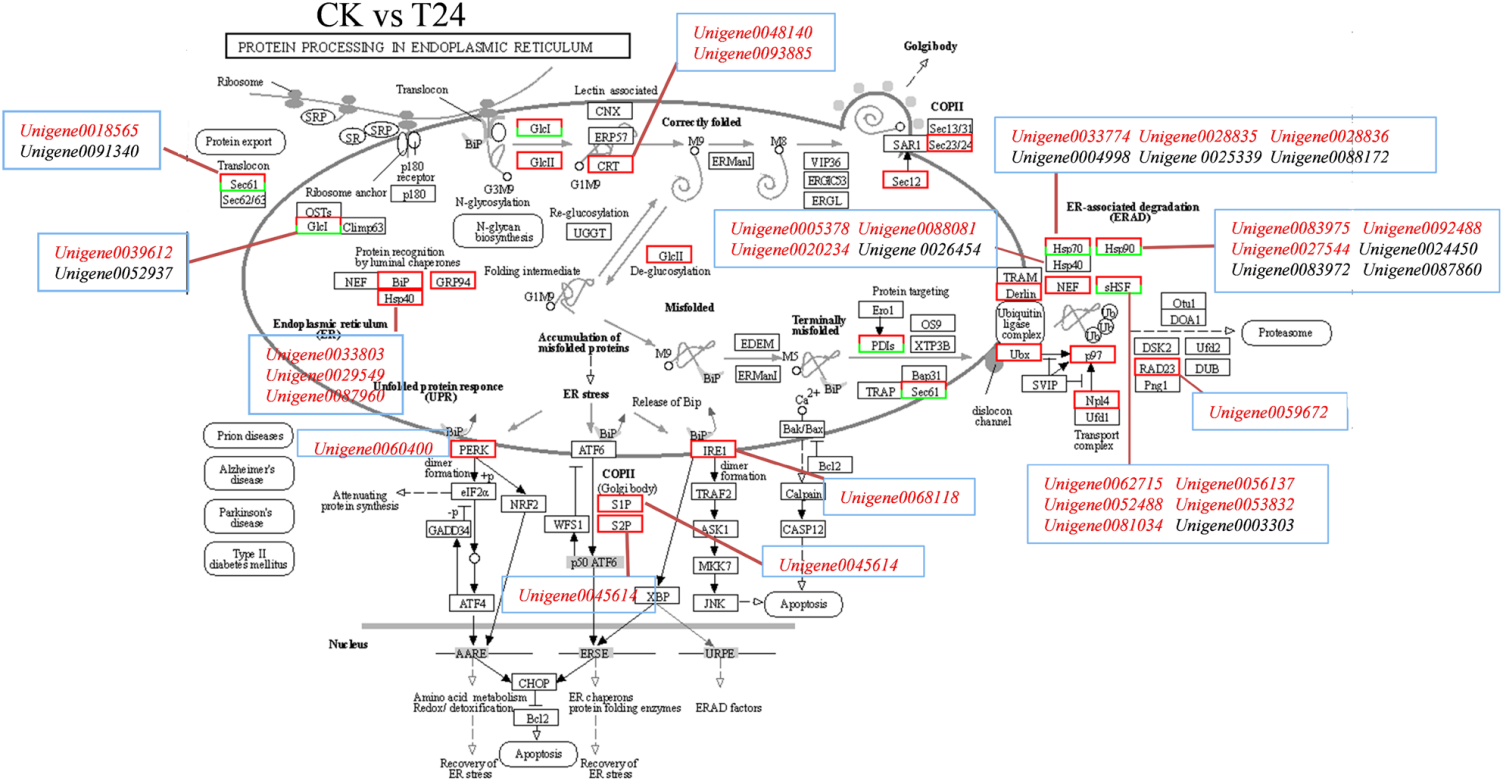
Pathway of processing pathways in the ER.

**Figure 12:**
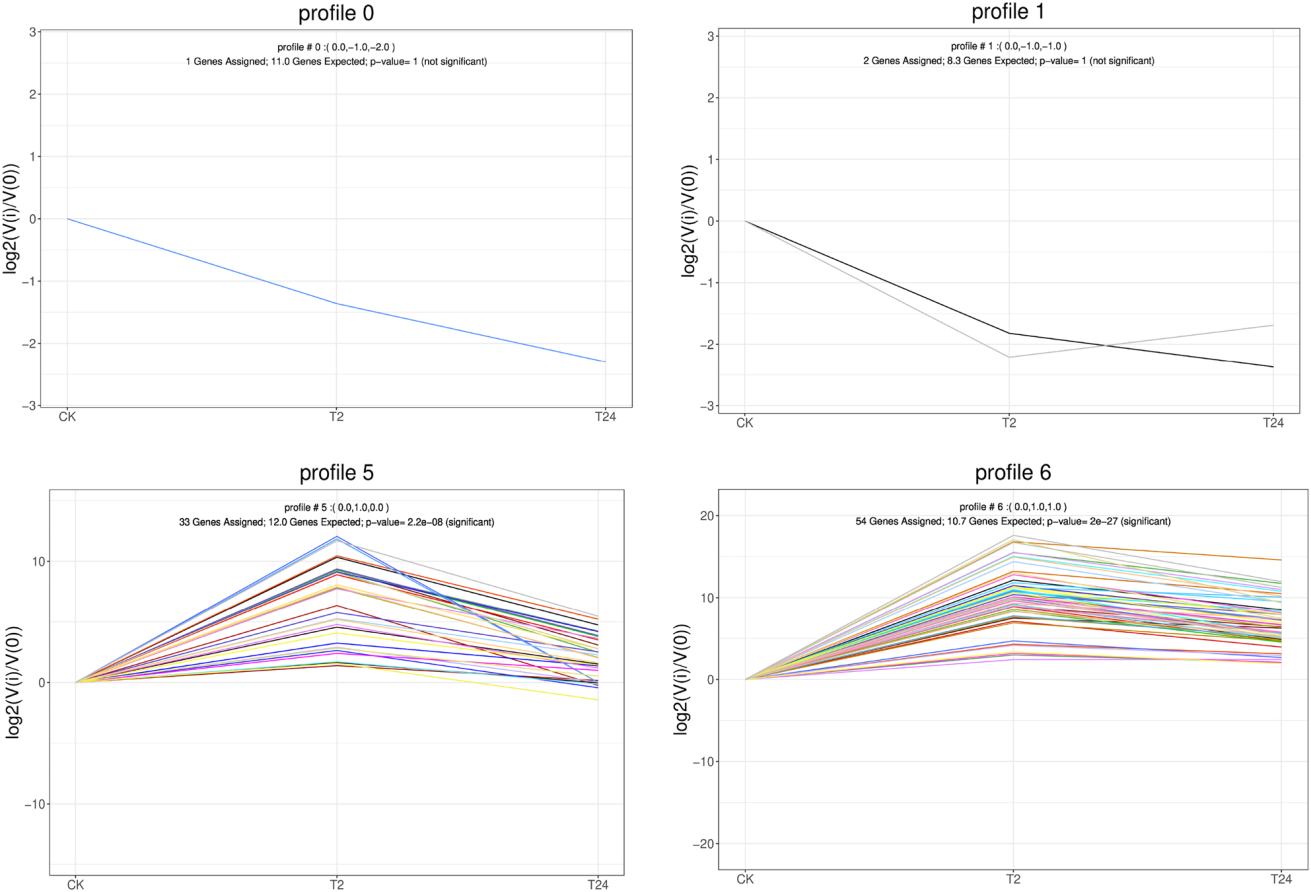
Expression patterns of HSP genes.

Heat stress can cause protein folding error, ER stress and cell death. Therefore, the ER plays an important role in plant response to high-temperature stress. In this study, after high-temperature stress, 155 and 125 DEGs were found after 2 and 24 h of high-temperature treatment in the protein processing in the ER (ko04141). Most of them were associated with heat stress, including HSP40, HSP70 and HS90 (Fig. 11, Table S4). In the CK- versus -T2 and CK -versus -T24 comparison groups, 90 and 71 heat shock protein (*HSP*) genes, respectively, were identified among the DEGs. Moreover, the expression levels of most *HSP* genes in CK and at 2 and 48 h showed a trend of first increasing and then decreasing (Fig. 12). These results suggest that garlic may rapidly respond to heat stress by regulating the expression of *HSP* and ER-related genes at the transcriptional level to clear misfolded proteins induced by heat stress.

### Plant hormone signal transduction-related genes under changes in heat stress expression

**Figure 13:**
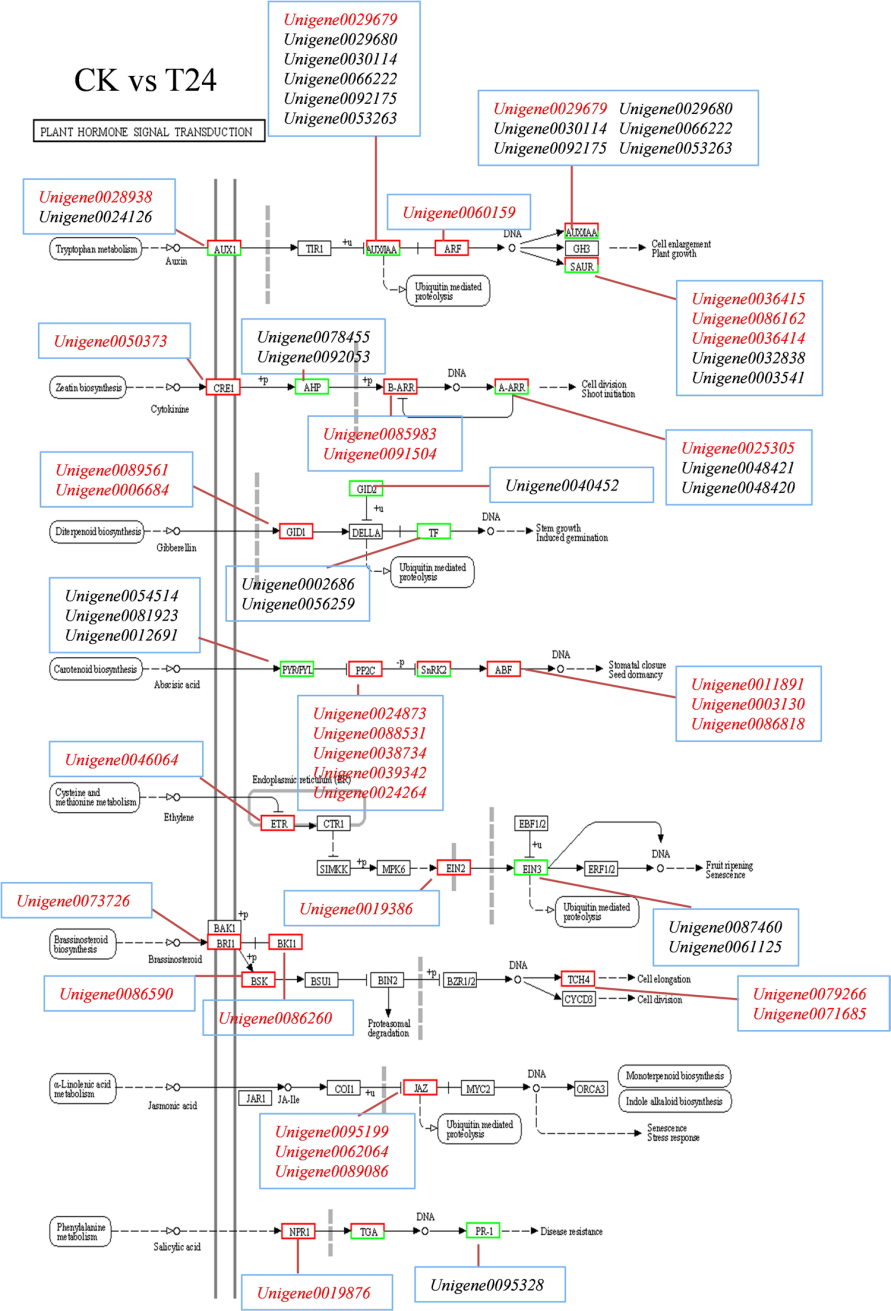
Plant hormone signal transduction pathway.

In the plant hormone signal transduction (ko04075) pathway, 63 and 66 DEGs were found after 2 and 24 h of high-temperature treatment, respectively. There were 20 genes affecting plant growth, including ABA, AUX, IAA, SAUR, and ARF, and most of these genes were downregulated in the late stage of heat stress. Among them, four ABF genes (*Unigene0090235, Unigene0030557, Unigene0060159,* and *Unigene0087890*) were upregulated after exposure to high temperatures for 2 and 24 h. Furthermore, there were nine genes affecting stomatal closure. The expression of *PYL* genes was downregulated after 24 h of treatment, and the expression of *PP2C* genes was upregulated after 2 and 24 h of treatment (Table 3). At the same time, under high-temperature stress, species DEGs related to plant signal transduction pathways were abundant, indicating that plant hormones play an important role in regulation under high-temperature stress (Fig. 13).

**Table 3:** Changes in DEGs in the KEGG pathway of plant hormone signal transduction at different time points during heat stress.

Code	Gene ID	Number of DEGs		Gene family names	Effect on plant growth
		2 h-63	24 h-66		
1	Unigene0001643	**+**	**−**	ABA2	Plant growth
2	Unigene0001589	**+**	**+**	ABA4	Plant growth
3	Unigene0001589	**+**	**−**	AUX22D	Plant growth
4	Unigene0030114	**−**	**−**	AUX22B	Plant growth
5	Unigene0070504	**−**	**−**	AUX22B	Plant growth
6	Unigene0084211	**+**	**−**	IAA4	Plant growth
7	Unigene0029680	**−**	**−**	IAA30	Plant growth
8	Unigene0029679	**−**	**+**	IAA26	Plant growth
9	Unigene0066222	**−**	**−**	IAA26	Plant growth
10	Unigene0092175	**−**	**−**	IAA25	Plant growth
11	Unigene0053263	**−**	**−**	IAA1	Plant growth
12	Unigene0078183	**−**	**−**	SAUR50	Plant growth
13	Unigene0032838	**−**	**−**	SAUR32	Plant growth
14	Unigene0036414	**+**	**−**	SAUR32	Plant growth
15	Unigene0036415	**+**	**−**	SAUR32	Plant growth
16	Unigene0080612	**−**	**+**	SAUR32	Plant growth
17	Unigene0090235	**+**	**+**	ABF19	Plant growth
18	Unigene0030557	**+**	**+**	ABF31	Plant growth
19	Unigene0060159	**+**	**+**	ABF11	Plant growth
20	Unigene0087890	**+**	**+**	ABF17	Plant growth
21	Unigene0066683	**+**	**−**	PYL8	Stomatal closure
22	Unigene0012691	**−**	**−**	PYL6	Stomatal closure
23	Unigene0054514	**−**	**−**	PYL4	Stomatal closure
24	Unigene0081923	**−**	**−**	PYL4	Stomatal closure
25	Unigene0063488	**−**	**−**	PYL2	Stomatal closure
26	Unigene0024873	**−**	**+**	PP2CA	Stomatal closure
27	Unigene0039342	**−**	**+**	PP2CA	Stomatal closure
28	Unigene0024164	**−**	**+**	PP2C51	Stomatal closure
29	Unigene0038734	**+**	**−**	PP2C06	Stomatal closure

### Quantitative real-time PCR (qRT-PCR) validation of differential gene expression under heat stress

**Figure 14:**
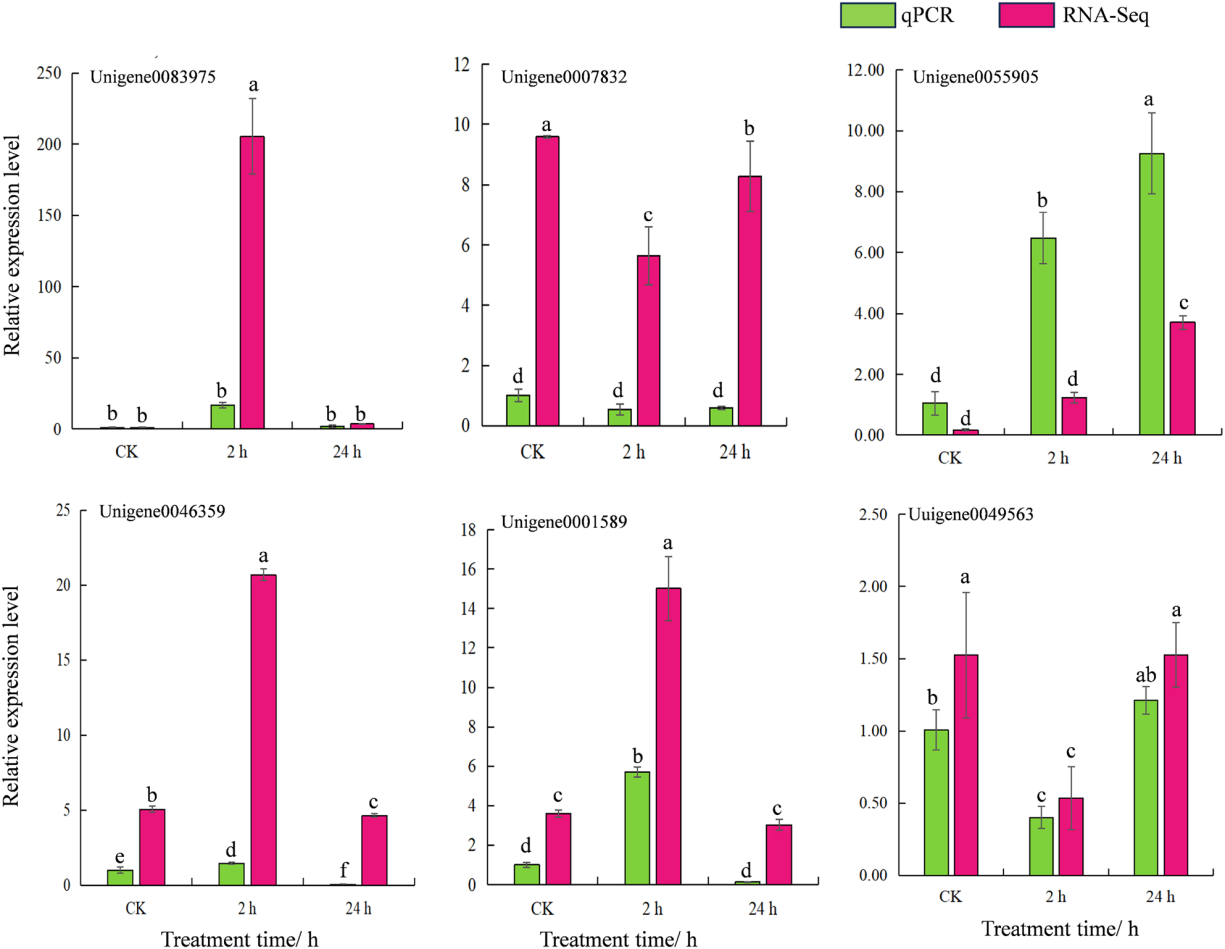
qRT-PCR validation of the expression levels of DEGs.

We used RT-qPCR to further verify the accuracy of the transcriptome data. Six unigenes were selected from the list of DEGs based on their potential functions: *Unigene0083975* (*HSP90-3*), *Unigene0007832* (*NDPK1*), *Unigene0046359* (*WRKY24*), *Unigene0055905 *(*CAT1*), *Unigene0001589 *(*AUX22D*), and *Unigene0049563* (*MYB5*). The analysis showed that although there were certain differences in the multiples of zero upregulation or downregulation of expression detected by RNA-seq and qRT-PCR, as shown in Fig. 14, the expression trend reflected by the results of qRT-PCR was consistent with the results of transcriptome sequencing, which may be caused by the different detection ranges and expression calculation methods of the two methods. The transcriptome sequencing data of garlic high-temperature stress were accurate and reliable.

### Models of the molecular mechanisms of garlic in response to heat stress

**Figure 15:**
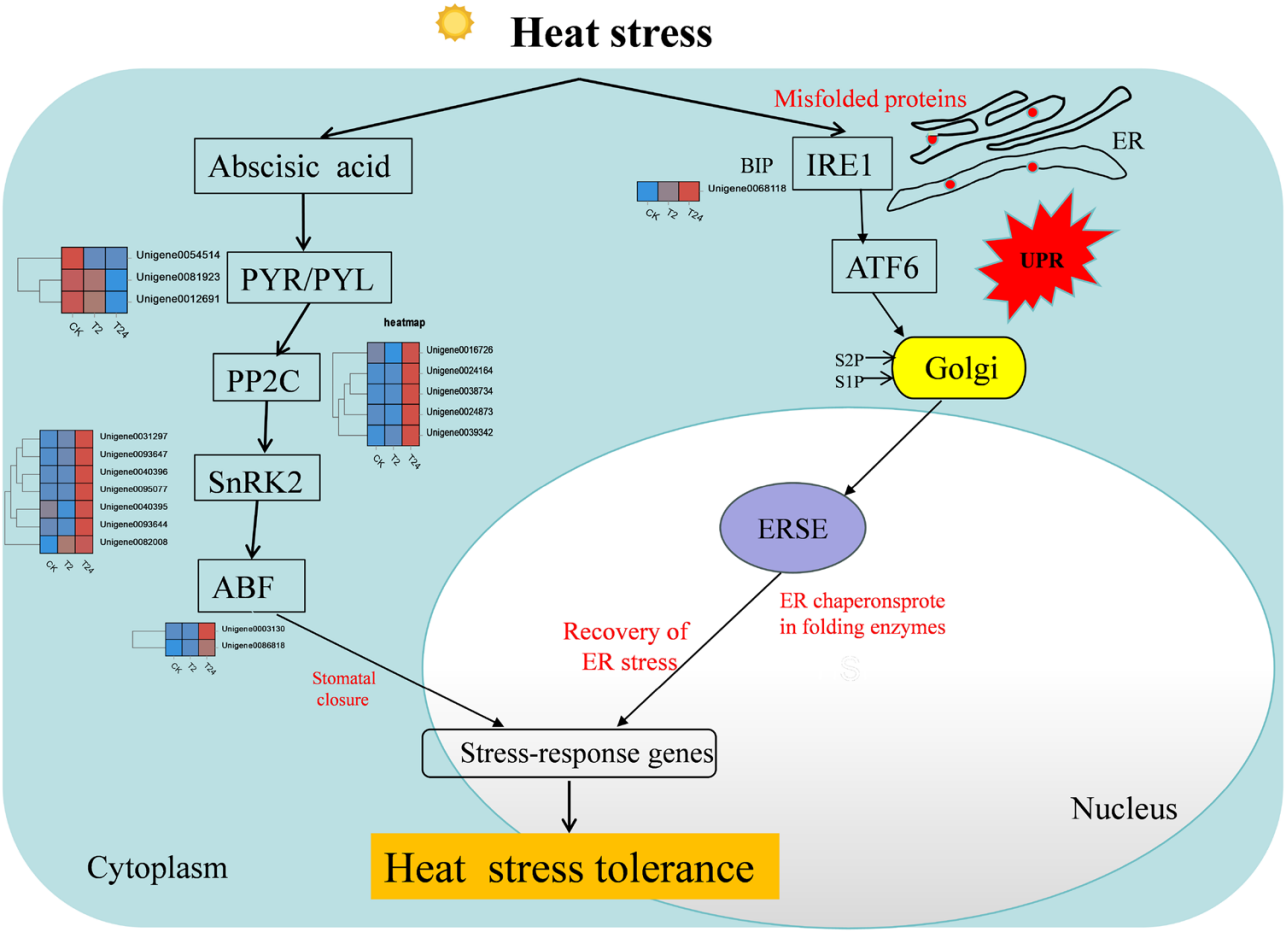
Schematic model of ABA signaling pathways and ER in the heat stress response of garlic.

Based on analysis of the transcriptome sequencing results, we developed a schematic model (Fig. 15). In the protein processing in the ER pathway, the expression of most HSPs is rapidly induced by high temperatures and may play an important role in preventing the formation of misfolded protein structures in garlic cells, as well as in rescuing aggregated or denatured proteins. In the plant hormone signal transduction pathway, ABA is an important hormone for regulating stress responses. After ABA bound to receptor protein PYR/PYL, the inhibition of kinase SnRK2 activity by phosphatase PP2C was relieved, and SnRK2 activity was activated to induce the plant stress response.

## Discussion

As an important environmental factor, temperature is involved in regulating many aspects of plant growth and development and has a very important impact on agricultural production^21,22^. Heat stress can lead to stalk elongation, early flowering and a serious decrease in crop yield^23^. Garlic leaves gradually withered and turned yellow after 24 h of exposure to high-temperature stress. POD activity increases rapidly to clear active oxygen species, and MDA accumulates continuously to destroy the cell membrane structure. Therefore, it is of great significance to study the mechanism of garlic coping with high-temperature stress for the cultivation of high-temperature-resistant varieties.

Studies have shown that plant responses to heat stress are carried out through a complex gene regulatory network^24^. In this study, RNA-seq was used to analyze the changes in the gene expression profiles of garlic treated at different times under high temperatures. In total, 12,727 and 10,343 DEGs were detected in response to heat stress at 2 and 24 h, respectively. Through the GO function of differential genes and the enrichment of the KEGG metabolic pathway, the primary metabolic processes, such as amino acid metabolism, carbohydrate metabolism and some secondary metabolic processes, began to be affected after 2 h of high-temperature stress. At the same time, the high-temperature stress response genes involved in the metabolic pathway of plant hormone signal transduction began to accumulate in the early 2 h of treatment. The results indicated that the stress signal reception and signal transduction processes of garlic were activated in the early stage of high-temperature stress and also began to regulate some primary and secondary metabolic processes.

Under abiotic stress conditions, the accumulation of unfolded or misfolded proteins in the ER can lead to ER stres^25^^.^ Li et al. also found that the upregulation of ER protein processing in maize was most significant after high-temperature stress, which was consistent with our research results^26^. In this study, KEGG enrichment analysis showed that the ER protein processing pathway was most enriched, indicating that ER stress occurs and triggers the UPR, which may play a central role in garlic leaves under heat stress. The ER luminal binding protein (BIP) is the core molecular chaperone that assists protein folding in the ER; upregulation of BIP gene expression can promote the folding of ER protein^27,28^. In protein processing in the ER pathway, ER stress mediated by inositol-dependent enzyme 1 (IRE1) is the driver of the early (1–3 h) heat stress response^29^. Under high-temperature stress, the *IRE1A* gene of garlic also significantly increased after 2 h of treatment, indicating that the IRE1A gene played a certain role in the early heat shock response of garlic. High-temperature stress can cause protein degeneration in organisms^30^. Heat shock protein (HSP) acts as a molecular chaperone to assist protein refolding, stabilization, intracellular transport, and degradation, prevent the accumulation of damaged proteins, and maintain the stability of the intracellular environment^31,32^_._ High-temperature stress transcripome analysis of quinoa showed that HSF transcription factors such as *LOC110702486*, *LOC110697083*, *LOC110729384*, *LOC110709409*, and *LOC11073445* were the core regulatory factors of transcription under high temperatures^33^. When plants are subjected to high-temperature stress, HSP accumulates a large amount of expression in a short time and participates in the stress response, but with the increase in treatment time, the stress response is weakened and HSP is degraded^5^. Transcriptome analysis showed that *HSP* genes were mainly concentrated in the protein processing pathways in the ER. Moreover, the expression levels of most *HSP* genes increased first and then decreased after 2 and 24 h of high-temperature treatment, which may be due to the short-term nature of HSP.

Plant endogenous hormones are very important regulatory factors in plant growth and development^34^. When plants are exposed to environmental stress, the synthesis, distribution and transport of endogenous hormones change significantly to activate the plant stress resistance mechanism^35^_._ In response to high-temperature stress, plant hormones, including ABA, IAA, AUX, SAUR, ARF, and PYL, are signaling compounds that regulate important aspects of growth, development, and environmental stress response^5^. Plants subjected to high-temperature stress can rapidly accumulate a large amount of ABA, which can bind to PYR/PYL (Pyrabactin resistance 1/Pyrabactin resistance 1-like) to inhibit PP2C (Type 2C protein phosphatase) activity, but snPK2 (Sucrosenon-fermenting 1-related protein 2) remains active and phosphorylates the downstream transcription factor ABF^36^. For example, Kim et al. found that overexpression of ABA transcription factor ABF3 can make plants resistant to high-temperature stress^37^. In this study, garlic may also form PYR-PP2C-SnRK2 signal transduction complex under high-temperature stress and then participate in the activation of ABA to initiate the mechanism of resistance to high temperature. The signal transduction pathway of auxin in response to high-temperature stress mainly includes transport inhibitor response1/auxin signaling F-box (TIR1/AFB), auxin/indoleacetic acids proteins (Aux/IAA), auxin response factors (ARFs) and other protein components^38^. Chen et al. found that the expression of many SbARFs in sorghum was upregulated by high-temperature stress, and SbARF17/24 accumulated in vascular tissue under high-temperature stress, indicating that SbARF may participate in the high-temperature response^39^. In this study, the expression levels of four *ABF* genes (*Unigene0090235*, *Unigene0030557*, *Unigene0060159*, and *Unigene0087890*) were upregulated at 2 and 24 h under heat stress. These DEGs may be key candidate genes related to auxin in garlic. At the same time, DEGs in plant signal transduction pathways were abundant under high-temperature stress. It shows that plant hormones play an important role in the regulation of high-temperature stress.

In this study, we screened and obtained important DEGs related to protein processing in the ER. At present, there are few transcriptome studies on abiotic stress in garlic seedlings. Therefore, a comparative analysis of transcriptomes based on different garlic varieties and growth stages may be of great significance in elucidating some common molecular mechanisms of garlic abiotic stress response.

## Materials and methods

### Plant materials and growth conditions

The garlic cultivar ‘Xusuan No. 6’ was used as the experimental material, and it was conserved at the Xuzhou Institute of Agricultural Sciences in Jiangsu Xuhuai Area. It was selected and the bulbs that had been released from dormancy full and undamaged were selected and then grown in a mixture of organic soil and vermiculite in a greenhouse (34° 27′ N, 117° 29′ E). The temperature of the culture in the artificial climate chamber was set to 25 °C, with a relative humidity of 30% and a photoperiod of 16 h of light and 8 h of dark. To ensure that the plants are not exposed to drought at high temperatures, after two weeks, the garlic seedlings were transplanted to a hydroponic tank with 1/2 Hoagland nutrient solution and maintained in this solution for six days. In addition, plants with consistent and robust growth were selected for high-temperature treatment (38 °C).The relative humidity of the air was set to 30%, and each treatment was repeated 3 times. At 0 (CK), 2 and 24 h of treatment, the upper leaves of the garlic plant were removed, frozen in liquid nitrogen and stored at − 80 °C for later use.

### Determination index

The activities of POD and MDA were determined according to the manufacturer’s instructions (Beijing Solarbio Technology Co., Ltd., Beijing, China). The chlorophyll content was determined using the ethanol extraction colorimetric method. In the presence of a small amount of quartz sand and calcium carbonate powder, leaf chlorophyll was extracted with 95% ethanol. The entire extraction process was carried out under dark conditions. The absorbance values at 665, 649, and 470 nm were determined using a spectrophotometer, with 95% ethanol serving as the control. The chlorophyll contents were then calculated based on these absorbance values. Each index was determined 3 times, and the average value was calculated using Excel 2016 software. The method was analyzed and compared with Duncan’s method using SPSS software.

### RNA extraction, library construction, and RNA-seq

Total RNA was extracted using a plant RNA extraction kit (Tiangen, Beijing, China) in accordance with the manufacturer’s instructions. The integrity of nucleic acid samples was tested by agarose gel electrophoresis. The purity of nucleic acid is determined by detecting OD value of nucleic acid by NanoDrop. The Agilent 2100 assay uses an RNA integrity value (RIN) to determine the quality of the RNA. Subsequently, messenger RNA (mRNA) was enriched using oligo (dT) beads, and the ribosomal RNA (rRNA) was removed using a Ribo-ZeroTM Magnetic Kit (Epicentre). Then, the enriched mRNA was fragmented into short fragments using fragmentation buffer and reverse transcribed into cDNA with random primers. Second-strand cDNA was synthesized by DNA polymerase I, RNase H, dNTP, and buffer. Then the cDNA fragments were purified with QiaQuick PCR extraction kit(Qiagen, Venlo, The Netherlands), end repaired, A base added, and ligated to Illumina sequencing adapters. The ligation products were size selected by agarose gel electrophoresis, PCR amplified, and sequenced using Illumina HiSeqTM 2500 by Gene Denovo Biotechnology Co. (Guangzhou, China). The construction of the library was carried out according to Yu’s method^40^.

### Transcriptome sequencing and de novo assembly analysis

To ensure that data quality does not affect subsequent assembly and analysis, the read data were further filtered through fastp^41^ (version 0.18.0) to produce high-quality clean reads. The parameters were as follows: (1) removing reads containing adapters; (2) removing reads containing more than 10% of unknown nucleotides (N); (3) removing low quality reads containing more than 50% of low quality (Q-value ≤ 20) bases.

### Analysis of differentially expressed unigenes

DESeq2 software was used to analyze the differential RNA expression between the two groups^42^. Genes were considered differentially expressed when the value of log_2_ Fold Change was > 2 or <  − 2 with an FDR value below 0.01 between the two groups. GO functions and KEGG pathway enrichment analysis of DEGs were conducted using the hypergeometric test by comparing them with the whole genome background. Gene Ontology (GO)^43^ is an international standardized gene functional classification system which offers a dynamic-updated controlled vocabulary and a strictly defined concept to comprehensively describe properties of genes and their products in any organism. The calculated p-value was gone through FDR Correction, taking FDR ≤ 0.05 as a threshold. GO terms meeting this condition were defined as significantly enriched GO terms in DEGs. KEGG is the major public pathway-related database^44^. The calculatedp-value was gone through FDR Correction, taking FDR ≤ 0.05 as a threshold. Pathways meeting this condition were defined as significantly enriched pathways in DEGs.

### Quantitative real-time PCR

Total RNA was extracted using a plant RNA extraction kit (Tiangen, Beijing, China) in accordance with the manufacturer’s instructions and cDNA was synthesized using a FastQuant RT Kit (Tiangen Biotech, Beijing, China). qRT-PCR was performed following the instructions of the SYBR Premix Ex Taq kit (Takara, Dalian, China). The quantitative primers were designed using Primer Premier 6.0; all primers are listed in Table 4. The reaction process consisted of 40 cycles of pre-denaturation at 95 °C for 10 min, followed by denaturation at 95 °C for 5 s and annealing/extension at 60 °C for 40 s. qRT-PCR analysis was performed on the CFX96 Real-Time PCR system (Bio-Rad). The PCR reaction system had a volume of 20 μL. The cDNA concentration of each PCR system was 3.33 ng/uL, the concentration of 0.4 μL positive quantitative primer and 0.4 μL negative quantitative primer were 100 umol/L 10 μL of SYBR Premix Ex Taq, and 7.2 μL of ddH_2_O. The relative gene transcription level was calculated according to the 2^−ΔΔCT^ method^45^. The data in this paper were analyzed using the least significant difference (LSD) test in SPSS 17.0 statistical software. We used actin gene amplification as a normalized internal control. Three biological replicates were performed, with three technical replicates for each biological replicate.

**Table 4:** Primer sequences for qRT-PCR.

Gene ID	Forward primer (5′ → 3′)	Reverse primer (5′ → 3′)
*Unigene0046359*	*CCAGGACGCAACAACCAACTCA*	*CGTGGCAGCCGAATCATTCTGT*
*Unigene0001589*	*GACTTGGTTTGCCTGGGATGGA*	*TGCCGTTTGCTTCTGTTTCAGT*
*Unigene0055905*	*TGCCAATACCACCTGCTGTACT*	*ACCTTGAGACGACTTGCCAACT*
*Unigene0049563*	*CGGAGCACCACACAATGAACC*	*AGCGACAGACACCAGAAGAGAA*
*Unigene0007832*	*CCTGGTACTATCCGTGGCGATT*	*CTGCCAGTTGGAAATGCCCTCT*
*Unigene0083975*	*TGTCGAGCAAGAAGACGATGGA*	*ACCGTCAGCATCAGCATCATCC*
*ACTIN*	*TGCTCTGGATTATGAACAGGAACTTGA*	*CAATCATTGAAGGCTGGAACAACACT*

### Statistical analysis

The graphs were generated using GraphPad Prism 6.0 software. The bars represent the mean values of the three biological replicates ± standard deviation.

### Ethical approval

All the plant experiments/protocols were performed with relevant institutional, national, and international guidelines and legislation.

### Supplementary Information

Supplementary Table S1.

Supplementary Table S2.

Supplementary Table S3.

Supplementary Table S4.

## Data Availability

The data supporting the findings of this study are available from the corresponding authors upon request. The raw sequences data was uploaded to Sequence Read Archive (https://submit.ncbi.nlm.nih.gov/subs/sra/).The accession numbers are uploaded and archived at SRA with accession PRJNA1071790.
