# Supplementary material for: Transcriptome sequencing of garlic reveals key genes related to the heat stress response
Source: Sci Rep. 2024 Jul 10;14:15956. doi: 10.1038/s41598-024-66786-4 (PMC11236982; doi:10.1038/s41598-024-66786-4)
Supplement: Supplementary file 1 — Supplementary Table S1. [file 41598_2024_66786_MOESM1_ESM.docx]

Table S1 Top 20 KEGG pathways in CK-vs-T2 group

| KEGG_A_class | Pathway | Gene  numbers | Pvalue | KEGG  Pathway |
| --- | --- | --- | --- | --- |
| Genetic Information Processing | Protein processing in  endoplasmic reticulum | 155 | 0.00000 | ko04141 |
| Organismal Systems | Plant-pathogen  interaction | 70 | 0.00086 | ko04626 |
| Environmental Information Processing | Plant hormone signal transduction | 63 | 0.00010 | ko04075 |
| Metabolism | Starch and sucrose metabolism | 44 | 0.00759 | ko00500 |
| Genetic Information Processing | Spliceosome | 72 | 0.01288 | ko03040 |
| Metabolism | Photosynthesis - antenna proteins | 7 | 0.02542 | ko00196 |
| Metabolism | Riboflavin metabolism | 9 | 0.02621 | ko00740 |
| Metabolism | Diterpenoid biosynthesis | 5 | 0.02890 | ko00904 |
| Environmental Information Processing | MAPK signaling pathway - plant | 39 | 0.03958 | ko04016 |
| Metabolism | Galactose metabolism | 18 | 0.05691 | ko00052 |
| Metabolism | Taurine and hypotaurine  metabolism | 6 | 0.05985 | ko00430 |
| Metabolism | Fatty acid elongation | 10 | 0.09524 | ko00062 |
| Metabolism | Zeatin biosynthesis | 6 | 0.09676 | ko00908 |
| Metabolism | Vitamin B6 metabolism | 6 | 0.11887 | ko00750 |
| Metabolism | Cyanoamino acid  metabolism | 19 | 0.11931 | ko00460 |
| Metabolism | Terpenoid backbone  biosynthesis | 16 | 0.13247 | ko00900 |
| Metabolism | Glycerolipid metabolism | 24 | 0.15495 | ko00561 |
| Metabolism | Pentose and glucuronate interconversions | 23 | 0.15738 | ko00040 |
| Metabolism | Amino sugar and nucleotide sugar metabolism | 36 | 0.16066 | ko00520 |
| Genetic Information Processing | Ubiquitin mediated proteolysis | 44 | 0.18658 | ko04120 |
