# Supplementary material for: Transcriptome sequencing of garlic reveals key genes related to the heat stress response
Source: Sci Rep. 2024 Jul 10;14:15956. doi: 10.1038/s41598-024-66786-4 (PMC11236982; doi:10.1038/s41598-024-66786-4)
Supplement: Supplementary file 2 — Supplementary Table S2. [file 41598_2024_66786_MOESM2_ESM.docx]

Table S2 Top 20 KEGG pathways in CK-vs-T 24 group

| KEGG_A_class | Pathway | Gene  numbers | Pvalue | KEGG  Pathway |
| --- | --- | --- | --- | --- |
| Genetic Information Processing | Protein processing in endoplasmic reticulum | 125 | 0.00000 | ko04141 |
| Metabolism | Phenylpropanoid biosynthesis | 38 | 0.00166 | ko00940 |
| Metabolism | Photosynthesis - antenna proteins | 10 | 0.00183 | ko00196 |
| Metabolism | Metabolic pathways | 717 | 0.00224 | ko01100 |
| Metabolism | Diterpenoid biosynthesis | 7 | 0.00333 | ko00904 |
| Metabolism | Flavone and flavonol biosynthesis | 5 | 0.00695 | ko00944 |
| Metabolism | Biosynthesis of secondary metabolites | 382 | 0.00904 | ko01110 |
| Environmental Information Processing | Plant hormone signal transduction | 66 | 0.00954 | ko04075 |
| Metabolism | Glutathione metabolism | 38 | 0.01156 | ko00480 |
| Metabolism | Riboflavin metabolism | 11 | 0.01518 | ko00740 |
| Metabolism | Taurine and hypotaurine metabolism | 8 | 0.01839 | ko00430 |
| Metabolism | Glycerolipid metabolism | 33 | 0.03489 | ko00561 |
| Metabolism | Fatty acid elongation | 13 | 0.04404 | ko00062 |
| Metabolism | Flavonoid biosynthesis | 14 | 0.04729 | ko00941 |
| Cellular Processes | Peroxisome | 28 | 0.04894 | ko04146 |
| Metabolism | Biosynthesis of unsaturated fatty acids | 11 | 0.05742 | ko01040 |
| Metabolism | Fatty acid metabolism | 34 | 0.06087 | ko01212 |
| Metabolism | Ubiquinone and other terpenoid-quinone biosynthesis | 18 | 0.06533 | ko00130 |
| Metabolism | Glycosphingolipid biosynthesis - globo and isoglobo series | 4 | 0.07454 | ko00603 |
| Metabolism | Carotenoid biosynthesis | 13 | 0.09277 | ko00906 |
