# Supplementary material for: Transcriptome sequencing of garlic reveals key genes related to the heat stress response
Source: Sci Rep. 2024 Jul 10;14:15956. doi: 10.1038/s41598-024-66786-4 (PMC11236982; doi:10.1038/s41598-024-66786-4)
Supplement: Supplementary file 3 — Supplementary Table S3. [file 41598_2024_66786_MOESM3_ESM.docx]

Table S3 Top 20 KEGG pathways in T2-vs-T24 group

| KEGG_A_class | Pathway | Gene  numbers | Pvalue | Pvalue |
| --- | --- | --- | --- | --- |
| Genetic Information Processing | Protein processing  in endoplasmic reticulum | 164 | 0.00000 | ko04141 |
| Metabolism | Metabolic pathways | 912 | 0.00003 | ko01100 |
| Metabolism | Glutathione metabolism | 50 | 0.00092 | ko00480 |
| Metabolism | Diterpenoid biosynthesis | 8 | 0.00226 | ko00904 |
| Metabolism | Biosynthesis of  secondary metabolites | 472 | 0.01155 | ko01110 |
| Metabolism | Riboflavin metabolism | 13 | 0.01174 | ko00740 |
| Metabolism | Porphyrin metabolism | 26 | 0.01480 | ko00860 |
| Metabolism | Flavone and flavonol biosynthesis | 5 | 0.01934 | ko00944 |
| Metabolism | Terpenoid backbone biosynthesis | 27 | 0.02241 | ko00900 |
| Metabolism | Cutin, suberine  and wax biosynthesis | 14 | 0.02547 | ko00073 |
| Metabolism | Fatty acid elongation | 16 | 0.02741 | ko00062 |
| Environmental Information Processing | MAPK signaling  pathway - plant | 58 | 0.03444 | ko04016 |
| Metabolism | Photosynthesis - antenna proteins | 9 | 0.03447 | ko00196 |
| Metabolism | Purine metabolism | 38 | 0.04545 | ko00230 |
| Metabolism | Glyoxylate and dicarboxylate metabolism | 43 | 0.05006 | ko00630 |
| Metabolism | Taurine and hypotaurine metabolism | 8 | 0.06590 | ko00430 |
| Metabolism | Anthocyanin biosynthesis | 2 | 0.07512 | ko00942 |
| Metabolism | Ubiquinone and other terpenoid-quinone biosynthesis | 21 | 0.08931 | ko00130 |
| Metabolism | Amino sugar and nucleotide sugar metabolism | 56 | 0.10817 | ko00520 |
| Metabolism | Glycine, serine and threonine metabolism | 37 | 0.10908 | ko00260 |
